# Patient experience after pelvic exenteration: chronic pain and quality of life

**DOI:** 10.1007/s00520-026-10848-y

**Published:** 2026-06-15

**Authors:** Olivia Louis, J. Young, C. Power, C. Atkin, C. Behrenbruch

**Affiliations:** 1 Department of Anaesthesia and Pain, Peter MacCallum Cancer Centre, 305 Grattan Street, Melbourne, VIC 3000 Australia; 2 Department of Critical Care, University of Melbourne, Parkville, Australia; 3 Department of Surgery, Peter MacCallum Cancer Centre, Melbourne, Australia

**Keywords:** Pelvic exenteration, Chronic pain, Disability, Pelvic cancer

## Abstract

**Background:**

Pelvic exenteration is a major surgery involving resection of the pelvic viscera and surrounding structures. Performed on patients with locally advanced or recurrent pelvic cancer, it is associated with high morbidity, persistent pain and low quality of life (QoL). This study aimed to determine the long-term prevalence of chronic pain and to characterise the pain and QoL experience in pelvic exenteration patients.

**Methods:**

A telephone survey was undertaken, utilising patient-reported outcome measures: the Chronic Pain Grade Scale (CPGS) to assess pain and the Short Form-12 (SF-12) to measure QoL. Historic and demographic data were retrieved from hospital records to capture potential pre-, peri- and post-operative determinants. Data was analysed using descriptive statistics, correlation and comparative tests.

**Results:**

This study comprised 48 individuals, up to 13 years post-pelvic exenteration. Pain prevalence was 75%, with most patients reporting no to low-intensity pain, without disability (54%). SF-12 scores varied; physical scores were significantly lower than population norms, while mental scores were preserved. Pain intensity and disability (CPGS sub-scores) were associated with lower QoL, a relationship that was consistent across pain grades.

**Conclusions:**

These findings indicate that the post-pelvic exenteration experience is characterised by reduced physical QoL and notable pain, highlighting the importance of early pain management optimisation. This research provides surgical candidates with authentic patient insight into life after surgery in a complex yet understudied population. Prospective longitudinal research is recommended to further examine patient trajectories and predictors of pain and poor QoL, enabling better targeted pain management.

**Supplementary Information:**

The online version contains supplementary material available at 10.1007/s00520-026-10848-y.

## Introduction

Pelvic exenteration (PE) is an extensive surgical procedure involving the en bloc removal of pelvic viscera and adjacent vascular, bony and nervous structures [1]. It is typically performed on individuals with locally advanced or recurrent pelvic malignancies, with notable survival benefits for curative intent [2]. Although mortality rates for exenterative surgery have declined over time [3], associated post-operative complications and morbidity rates remain high with almost 30% of patients experiencing a major post operative complication, most frequently intrabdominal abscesses [4]. Due to complexity of the surgery and the associated complications, many patients report acute post-surgical pain, which often persists beyond 3 months, evolving into chronic pain.

Despite high rates of initial acute post-operative pain, there is limited literature examining patients long-term pain experience following pelvic exenteration. Previous research has indicated that pain scores remain as high at 12 months post-operatively [5], with few studies extending beyond this follow-up period. Chronic pain is a known predictor of poor quality of life (QoL). The existing research on QoL trajectories in patients following pelvic exenteration is conflicting, often with short follow up periods. Some studies have shown a return to pre-operative quality of life within 2 years of surgery [2, 3, 6–8] while other studies report a sustained decline in quality of life up until mortality in palliative settings [2].

In select patients undergoing PE, such as in locally advanced rectal cancer, 5-year survival rates can be as high as 30% [9]. Therefore, understanding the duration and prevalence of chronic pain and the impact on quality of life is critical for developing strategies for identifying and improving patients experience of pain in the longer term and therefore overall cancer survivorship [10]. The goal of this study is to determine the current prevalence of chronic pain in a population of patients post–pelvic exenteration including the long-term characteristics of the pain and quality of life.

## Methods

This study was a multi-modal, single-centre, cross-sectional study examining the prevalence and characteristics of chronic pain and quality of life in patients who had undergone a pelvic exenteration surgery in the last 20 years at the Peter MacCallum Cancer Centre, a quaternary oncology hospital in Melbourne, Australia. The project involved a 30-min telephone survey that included validated patient-reported outcome measures and exenteration-specific questions to generate a point-in-time analysis of current characteristics. In addition, retrospective data was collected from patient medical records to identify potential contributing factors to the development of chronic pain as well as factors that could be managed early to potentially prevent the development of chronic pain.

Patients were identified from surgical and pain team electronic databases and considered eligible for inclusion if they were documented as having undergone any variation of pelvic exenteration during this period including ‘exenteration’, ‘anterior exenteration’, ‘posterior exenteration’ and ‘total exenteration’. The earliest pelvic exenteration captured by the electronic records was dated 24/11/2011. Physical records were not screened for additional earlier cases due to feasibility constraints.

Total exenteration was defined as removal of the bladder, colon and reproductive organs, anterior exenteration involved the removal of the bladder with or without reproductive organs, while posterior exenteration involved the removal of the colon with or without reproductive organs.

Patients were excluded if they were under 18 years of age at the time of surgery, required an interpreter’s assistance or had undergone PE within 3 months of the study period (1/3/2025) as insufficient time had elapsed for any surgical-related pain to qualify as chronic. Patients who were deceased or uncontactable were also excluded from the analysis.

Sample size was restricted by the pool of surviving eligible patients. No pre hoc power analyses were conducted as the analysis was based on the census of all available cases.

The primary measurable endpoint was the current prevalence of chronic pain, as reported by patients during the telephone interview. Secondary outcomes included pain severity and QoL as scored by the Chronic Pain Grade Scale (CPGS) and 12-item Short-Form Survey (SF-12), respectively. The CPGS [11] is a 7-point scale utilised to categorise overall pain severity as a grade. The CPGS grade is based on two sub-scores, characteristic pain intensity (sum of current, worst and average pain intensity on a 0–10 scale) and disability (pain interference with daily activities on a 0–10 scale). QoL was measured using the SF-12 [12], a 12-item generic QoL questionnaire that examines both physical and mental dimensions of QoL. The SF-12 score was selected over the longer SF-36 to minimise test fatigue for participants, while retaining sufficient predictive validity [13]. Other relevant clinical factors were recorded to identify potential predictors of pain outcomes.

Data was primarily collected via telephone questionnaire with participants (Appendix 1). In addition to the two validated scores, the survey included questions on current clinical management and pelvic exenteration-specific factors. Patient demographics and other relevant historic information was collected from ‘EPIC’, the electronic medical record system utilised at the study site. Data and outcomes were collected and stored in a REDCap database system managed and hosted by PMCC to ensure secure data management.

The ‘IBM Statistical Package for Social Sciences’ was utilised for statistical purposes. Descriptive statistics were performed to summarise patient demographics, patient-reported outcome measures and other documented variables. Categorical data was reported as frequencies, parametric outcomes were expressed as means and standard deviations (SD), while non-parametric data was expressed as medians and interquartile ranges (IQR). The CPGS and SF-12 scores were reported according to the scoring manual for each questionnaire, respectively [11, 12]. Appropriate *t*-tests, correlations and odds ratios were performed for further analysis with 95% confidence intervals where appropriate. As there was minimal missing data (3.50%), multiple imputation was deemed unnecessary.

This study received approval from the PMCC Human Research Ethics Committee and was conducted in accordance with the NHMRC National Statement on Ethical Conduct in Human Research (2024), the World Medical Association Declaration of Helsinki (2024) and the ICH Guideline for Good Clinical Practice (2024). In addition, this study received governance authorisation and site-specific assessment approval prior to study commencement. The HREC number for this study is 113,381. Clinical trial number not applicable.

Informed and verbal consent was provided by all included participants prior to the commencement of the telephone survey.

## Results

**Fig. 1:**
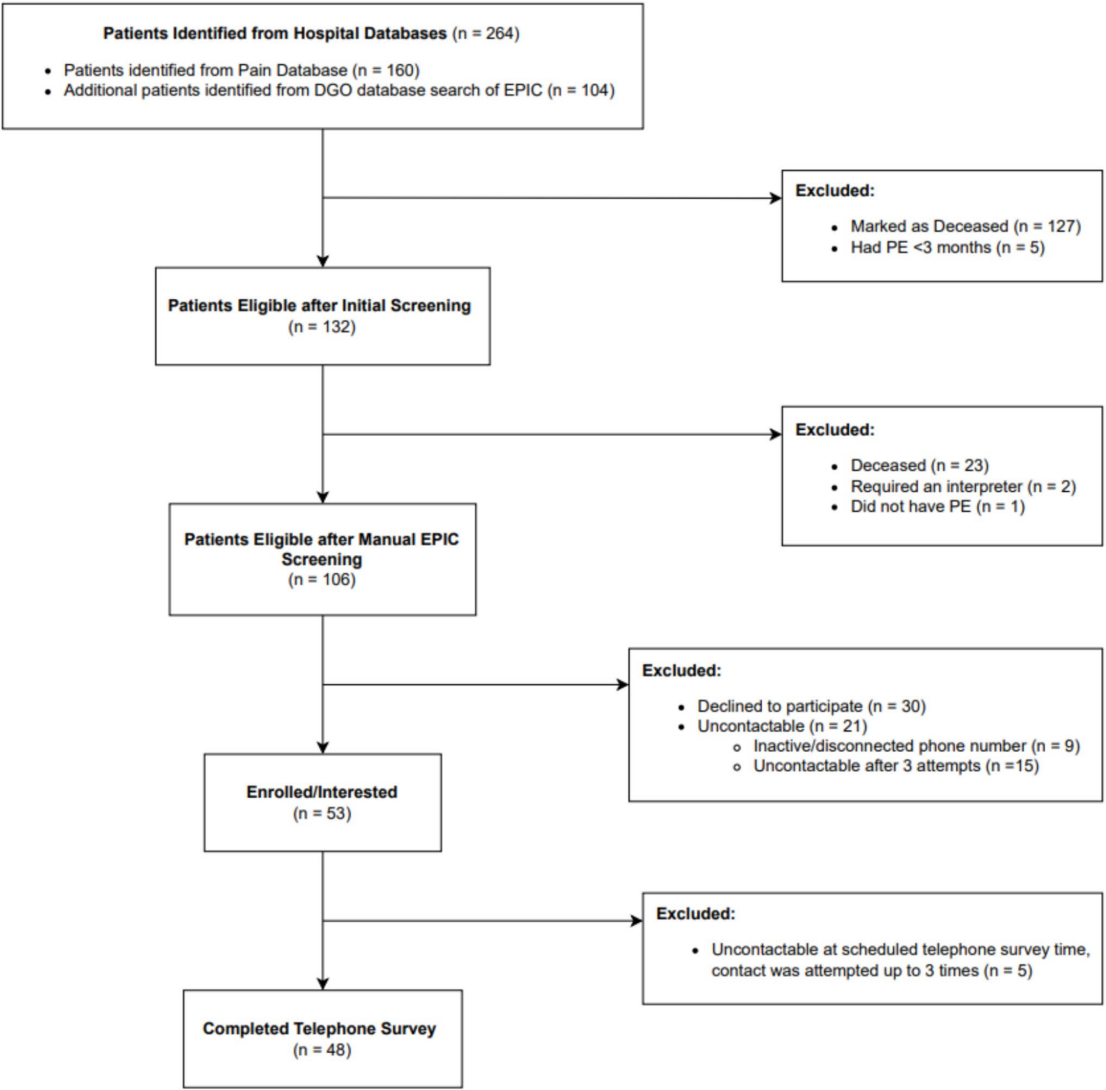
Patient flow diagram for identification, screening and recruitment

Of the 106 eligible patients after screening, 53 (50%) consented to participate. A final number of 48 (46%) completed the telephone survey and were included in the final analysis. The screening and recruitment process is outlined below in the patient flow diagram (Fig. 1). Patients declined to participate for a myriad of reasons including lacking time to complete the telephone interview, having no pain, currently undergoing other medical treatments and wishing to avoid revisiting negative experiences.

### General characteristics and peri- and post-operative factors

Table 1 summarises the patient demographics of this pelvic exenteration cohort. The mean age of participants was 63.69 ± 14.67, with ages ranging from 24 to 91 years. A majority of patients (75%) had a rectal cancer diagnosis, with most (81%) receiving adjunctive chemoradiotherapy. The mean time elapsed since pelvic exenteration was 5.79 ± 3.48 years, indicating that the study effectively captured a long-term post-operative patient pool.

**Table 1:** Patient demographics

		Mean ± SD	Median (IQR)	*N* (%)
Current age (years)		63.69 ± 14.67		
Weight (kg)			78.60 (33.60)	
Gender	Male			34 (71)
	Female			14 (29)
Preoperative history	Depression			16 (33)
	Anxiety			6 (13)
	Chronic pain			10 (21)
Trauma				16 (33)
	Physical			3 (6)
	Psychological			15 (31)
Substance use disorder				11 (23)
	Alcohol			5 (10)
	Cannabis			3 (6)
	Opioids			3 (6)
	Amphetamines			2 (4)
Smoking status	Never			29 (60)
	Current			6 (13)
	Ex-smoker			13 (27)
Cancer diagnosis	Primary—rectal			26 (54)
	Recurrent—rectal			9 (19)
	Primary—other			7 (15)
	Recurrent—other			6 (13)
Adjunctive therapy (neoadjuvant, intraoperative and adjuvant)	None			3 (6)
	Chemotherapy			3 (6)
	Radiotherapy			3 (6)
	Both			39 (81)
Time since surgery (years)		5.79 ± 3.48		

*N* = 48. *SD* standard deviation, *IQR *interquartile range

### Peri- and post-operative factors

Perioperative and post-operative factors up until current day are represented in Table 2. Most patients (96%) underwent PE as part of their direct cancer treatment, with two patients undergoing pelvic exenteration for other pelvic comorbidities including complications from previous surgery. Total pelvic exenteration was the most common procedure performed (83%), with 98% of patients achieving complete R0 resection (microscopically-negative tumour margins). The number of patients undergoing resection of adjacent structures was variable. All patients received patient-controlled analgesia (PCA) intravenously (using morphine or fentanyl). Many received an epidural (35%) and intrathecal morphine (39%), with 35% of patients having a spinal nerve block. There was significant variation in operation lengths and estimated blood loss as indicated, likely attributed to the complexity and diversity in surgeries conducted.

**Table 2:** Peri- and post-operative factors

		Mean ± SD	Median (IQR)	*N* (%)
Surgical indication	Cancer treatment			46 (96)
	Other			2 (4)
Surgical extent	Total			40 (83)
	Posterior			5 (10)
	Anterior			3 (6)
Resection margin	R_0_			47 (98)
	R_1_			1 (2)
	R_2_			0 (0)
High-complexity exenteration^a^				41 (81)
	Bony/sacrectomy			12 (25)
	Nerves			9 (19)
	Vascular			35 (73)
Flap reconstruction				34 (71)
Flap type	ALT			5 (10)
	IGAM			28 (58)
	VRAM			4 (8)
Perioperative analgesia	Spinal/nerve block			17 (35)
	Epidural			45 (94)
	Intravenous morphine (or equivalent)			48 (100)
	Local anaesthetic			39 (81)
Operation length (min)		619.23 ± 156.37		
Estimated blood loss (mL)			1200.00 (2000.00)	
Inpatient stay length			22.50 (11.00)	
Surgical/inpatient complications			2.00 (2.00)	
Return to theatre				18 (38)
Significant pelvic events since	Pelvic collection			16 (33)
	Hernia			22 (46)
	Recurrent UTI/urosepsis			19 (40)
	Small bowel obstruction			12 (25)
	Renal/urological complications			18 (38)
	Metastasis and/or Recurrence			11 (23)
Pelvic surgery since				32 (67)

*N* = 48. *SD* standard deviation, *IQR *interquartile range, *ALT* anterolateral thigh, *IGAM *inferior gluteal artery myocutaneous, *VRAM*  vertical rectus abdominis myocutaneous, *UTI *urinary tract infection

^a^High-complexity exenteration refers to procedures involving resection of adjacent anatomical structures [14]

Post-operatively, the median inpatient length of stay was 22.50 (11.00) days, with 38% of patients returning to theatre during their admission. Common ongoing comorbidities were hernias (46%), recurrent UTIs/urosepsis (40%) and pelvic collections (33%). Since the original pelvic exenteration, 67% of patients had an additional pelvic-related surgery to alleviate pain, comorbidities and recurrence. Several patients (23%) were diagnosed with recurrent or metastatic cancer post-operatively.

### Pain and management

Pain characteristics for the cohort are summarised in Table 3. The prevalence of pelvic-related pain in this cohort was 75%, with the average duration of persistent pain being 6.10 ± 3.55 years. Burden, as defined by the CPGS, represented an estimate for the total number of days participants experienced pain over the previous 6 months (out of 150 days). The median pain burden was 0.00, suggesting that most patients experienced intermittent pain. The most common sites of pain were the back (29%) followed by the peristomal (27%) and abdominal regions (23%).

**Table 3:** General pain characteristics

		Mean ± SD	Median (IQR)	*N* (%)
Prevalence				36 (75)
Duration (years)		6.10 ± 3.55		
Burden (days)			0.00 (150.00)	
Site	Donor site			11 (23)
	Flap			2 (4)
	Back			14 (29)
	Legs			10 (21)
	Feet			11 (23)
	Abdomen			12 (25)
	Peristomal			13 (27)
	Anal/rectum			10 (21)
	Penis (and testicles)			4 (8)
Side	Unilateral			15 (42)
	Bilateral			21 (58)
Type (sensation)	Shooting			14 (29)
	Muscular			16 (33)
	Painful to touch			5 (10)
	Dysesthesia			7 (15)
	Aching			20 (42)

*n* = 36. *SD* standard deviation, *IQR* interquartile range

The severity of interference in daily life and activities is described in Table 4. Patients reported that pain had the greatest effect on daily fatigue, with 36% of patients reporting a severe impact. Most patients reported no effect on sexual function, but the questions did not capture complete loss of sexual function and sensation which was expressed by most patients during the phone interview.

**Table 4:** Pain interference with daily life

	Nil	Mild	Moderate	Severe
	*N* (%)	*N* (%)	*N* (%)	*N* (%)
Sexual activity	31 (86)	1 (3)	1 (3)	3 (8)
Stoma care	24 (67)	3 (8)	8 (22)	1 (3)
Mood	7 (19)	12 (33)	10 (28)	7 (19)
Fatigue	8 (22)	10 (28)	5 (14)	13 (36)
Mobility	9 (25)	6 (17)	10 (28)	11 (31)

*N* = 36

Pharmacological management based on discharge summaries and current patient utilisation is summarised in Table 5. At discharge, 90% of patients were prescribed more than five medications, meeting the criteria for polypharmacy. Of these, 63% exhibited analgesic polypharmacy, defined as the use of three or more concurrent analgesic medications. In contrast, 20% reported current analgesic polypharmacy during the telephone survey. It is important to note that polypharmacy was not calculated for other medications as there was a lack of clarity in patient reported medication descriptions. At discharge, 85% of patients (with discharge medications listed) were prescribed opioids with a mean oral morphine equivalent (OME) of 68 mg, with only 25% reported current opioid utilisation. Other common medications at discharge were anti-neuropathics (36%), paracetamol (90%) and ondansetron (41%). Currently, patients predominantly utilise paracetamol (69%) and nonsteroidal anti-inflammatory drugs (NSAIDs) (31%) as required for pain management. Patients were also asked to estimate the percentage of relief received from current analgesic medications; the median relief was 65%. Anti-hypertensives and psychiatric medications were utilised by a relatively consistent number of patients across time points.

**Table 5:** Pharmacological management

	At discharge (*n* = 40)		Current (*n* = 48)	
	Mean ± SD	*N* (%)	Median (IQR)	*N* (%)
OME (mg)	67.57 ± 47.82		0.00 (0.00)	
Opioid tolerant (> 60 mg)		16 (40)		2 (4)
Opioids^a^		33 (85)		12 (25)
Anti-neuropathics		14 (36)		9 (19)
Paracetamol		35 (90)		33 (69)
NSAIDs		9 (23)		15 (31)
Ondansetron		16 (41)		0 (0)
Anti-hypertensives		16 (41)		15 (31)
Psychiatric		13 (33)		11 (23)
Medicinal cannabis		0 (0)		1 (2)
Other medications		40 (100)		32 (67)
Analgesic polypharmacy (> 3 medications)		25 (63)		9 (19)
Polypharmacy (> 5 medications)		36 (90)		–
Pain relief (%)		–	65.00 (60.00)	–

*SD* standard deviation, *IQR* interquartile range, *OME* oral morphine equivalent, *NSAIDs* nonsteroidal anti-inflammatory drugs

^a^Opioids included codeine, morphine, tramadol, tapentadol, oxycodone, buprenorphine and fentanyl

Current non-pharmacological management of pain is presented in Table 6. Most patients were currently engaged with their surgical team (77%) and their GP (92%), with several (17%) seeing a pain specialist. One third of patients had active allied health involvement and 19% employed non-pharmacological pain management strategies.

**Table 6:** Non-pharmacological management

			Median (IQR)	*N* (%)
Non-pharmacological pain management	TENS			2 (4)
	SCS			1 (2)
	Remedial massages			9 (19)
Current specialists involved			2.00 (2.00)	
	Allied health	Physiotherapist		15 (31)
		Osteopath		1 (2)
		Chiropractor		3 (6)
		Occupational therapist		4 (8)
	Clinical health	Colorectal surgeon		37 (77)
		Pain specialist		8 (17)
		Palliative care		2 (4)
		GP		44 (92)
		Psychiatrist		4 (8)

*N* = 48. *IQR* interquartile range, *TENS *transcutaneous electrical nerve stimulator, *SCS *spinal cord stimulator

The patient-reported outcome measure scores of the CPGS and SF-12 are detailed in Table 7. Both the CPGS and SF-12 scores varied across the cohort, as evidenced by wide IQRs and a large SD for the physical score. Although 75% of patients reported pain, patients were well distributed across the five pain grades, with most patients having zero to low pain intensity (54%). Physical QoL was low at a mean of 39.67 ± 11.89, whereas mental scores appeared relatively preserved at a median of 51.93 (14.21).

**Table 7:** Pain and QoL scores

		Mean ± SD	Median (IQR)	*N* (%)
Characteristic pain intensity			38.33 (53.34)	
Disability score			6.70 (56.65)	
CPGS pain grade	**Grade 0**—None			12 (25)
	**Grade I**—Low-intensity pain, without disability			14 (29)
	**Grade II—**High-intensity pain, without disability			6 (13)
	**Grade III**—Moderately limiting			5 (10)
	**Grade IV—**Severely limiting			11 (23)
SF-12 score	Physical component	39.67 ± 11.89		
	Mental component		51.93 (14.21)	

*N* = 48. *SD* standard deviation, *IQR *interquartile range

The physical and mental scores for the pelvic exenteration cohort were then compared to the population norms included in the original SF-12 scoring manual [12] and are presented in Supplementary Table 1. Physical scores were found to be significantly lower than population norms (*p* < 0.001), whereas mental scores were not (*p* = 0.073).

### The relationship between pain and quality of life

Spearman’s rank-order correlations were conducted to examine the relationship between pain intensity, disability, mental and physical QoL as presented in Table 8. Pain intensity was shown to be associated with an increase in disability (*p* < 0.001). Pain intensity and disability were both associated with a reduction in physical (*p* < 0.001; *p* < 0.001) and mental QoL scores (*p* = 0.045; *p* = 0.003).

**Table 8:** Spearman’s rank-order correlations between pain and QoL outcomes

	1	2	3
Characteristic pain intensity	–		
Disability score	0.67^**^ [0.45, 0.84]	–	
Physical score	− 0.64^**^ [− 0.80, − 0.43]	− 0.79^**^ [− 0.88, − 0.65]	–
Mental score	− 0.29^*^ [− 0.56, 0.02]	− 0.42^**^ [− 0.65, − 0.14]	0.28 [0.01, 0.53]

**p < 0.050. *p < 0.010. *ρ* = Spearman’s rank-order correlation. Bootstrapped bias-corrected and accelerated 95% confidence intervals were calculated using 1000 samples and are shown in square brackets

**Fig. 2:**
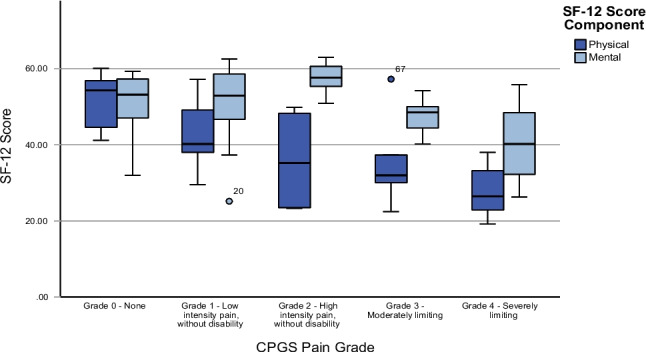
Comparison of PCS and MCS across the CPGS pain grades

To corroborate the findings of the correlation analysis, group differences across pain grades were assessed for physical and mental quality of life using comparative tests as displayed in Fig. 2. Both physical (*p* < 0.001) and mental scores (*p* = 0.010) were shown to differ significantly across pain groups. These results, presented in Fig. 2, suggest that physical and mental health declined as pain grade increased.

### Prognostic factors of pain

To identify potential prognostic factors for pain, odds ratios were calculated for various demographic, surgical and post-surgical variables, which are presented in Table 9. These factors were selected based on recommendation by the surgical team, previous pelvic exenteration literature [5, 10, 15] and well-established risk factors of chronic pain. Across the factors analysed, a significant association was identified between post-operative cancer recurrence or metastasis and the likelihood of experiencing pain (*p* = 0.044), with all individuals in this subgroup experiencing pain. Cancer recurrence in a pre-operative setting was also associated with the largest increased odds of pain (OR = 7.00), while surgical indications other than cancer treatment were associated with reduced odds (OR = 0.22); both of these factors approached statistical significance (*p* = 0.073; *p* = 0*.*059).

**Table 9:** Potential pre-, peri- and post-operative predictors of pain presence

Predictor	Odds ratio	95% CI	*p*
Pre-operative factors			
Gender (male/female)	2.50	[0.47, 13.27]	0.465
Age at surgery (years)	0.99	[0.94, 1.04]	0.574
Rurality (major city/regional)	0.45	[0.11, 1.76]	0.324
Cancer type (rectal/other)	1.15	[0.26, 3.49]	1.000
Cancer recurrence (primary/recurrent)	7.00	[0.81, 60.33]	0.073
Chemotherapy (N/Y)	1.39	[1.15, 1.67]	0.312
Radiotherapy (N/Y)	1.60	[0.25, 10.07]	0.631
Psychological distress (N/Y)	1.70	[0.39, 7.40]	0.725
Chronic pain (N/Y)	1.43	[0.26, 7.89]	1.000
History of trauma (N/Y)	3.18	[0.61, 16.72]	0.289
Peri-operative factors			
Surgical indication (cancer treatment/other)	0.22	[0.13, 0.38]	0.059
Extent of exenteration (total/partial)	1.00	[0.17, 5.77]	1.000
Bony resection (N/Y)	4.84	[0.56, 42.24]	0.247
Nerve resection (N/Y)	1.21	[0.21, 6.79]	1.000
Flap reconstruction (N/Y)	0.76	[0.17, 3.35]	1.000
Surgical/inpatient complications	1.32	[0.80, 2.17]	0.276
Post-operative factors			
Time since surgery	1.25	[0.92, 1.71]	0.156
Hernia (N/Y)	2.00	[0.51, 7.84]	0.505
Urosepsis (N/Y)	0.57	[0.15, 2.12]	0.501
Pelvic collections (N/Y)	1.70	[0.39, 7.40]	0.725
Metastasis/recurrence (N/Y)^a^	N/A	N/A	0.044
Surgical interventions since (N/Y)	1.62	[0.42, 6.26]	0.500
ED presentations since	1.22	[0.92, 1.63]	0.172
Current weight (kg)	0.98	[0.95, 1.02]	0.365
Current opioid utilisation (N/Y)	4.84	[0.55, 42.24]	0.247

*N* = 48. Fischer’s exact test was used to calculate significance for categorical variables, whereas Wald test was used for the continuous variables. 95% CI = 95% confidence intervals

^a^Odds ratios and 95% CI could not be estimated as all patients with metastasis/recurrence reported pain

## Discussion

### Pain prevalence and presentation

This study identified a high prevalence of pain (75%) in patients up to 13 years post-pelvic exenteration, highlighting the persistence of pain in a long-term setting.

Pain severity varied across pain grades, with most patients (29%) reporting low-intensity pain, without disability. The median disability score was low at 6.70 (56.65), suggesting that pain presence did not always translate into functional limitations. Our study showed more favourable pain outcomes compared to previous research [5, 10], potentially indicating improvements in surgical techniques, early optimisation and postoperative management. The single-centre design may limit the generalisability of these findings, as the study was conducted at an oncology hospital with a relative high frequency of and specialisation in pelvic exenterations. The heterogeneity of pain severity in this cohort reflects the complexity of pelvic exenteration cohorts, comprising subgroups with distinct medical histories and post-surgical trajectories. Such variation supports the need for individualised multidisciplinary and multimodal treatments. Clinical heterogeneity also complicates the ability to generate predictable outcomes for these patients because of covariates.

Pain duration experienced by patients was long (mean ± SD = 6.1 ± 3.5 years), highlighting the need for ongoing survivorship care beyond standard follow-up. Pain onset typically coincided with surgery, with 28% of patients reporting pre-operative chronic pain, supporting previous findings [18]. The timing of pain onset suggests that the procedure itself and other perioperative factors likely drive pain outcomes. The median pain burden of 0.00 (150.00) indicates that patients either experienced episodic pain or that pain severity did not constitute interference, supported by the positive skew in pain grade distribution (Table 7).

Types and sites of pain also varied across the cohort, further complicating management and supporting the need for patient-specific approaches. Previous studies have not examined pain in this level of detail, instead employing a standardised pain score that may not capture the true heterogeneity of the pain experience.

### Quality of life and the impact of pain

QoL was also measured to better capture the physical, emotional and social impact of surgery. Both mental and physical QoL varied across the cohort. Physical scores were determined to be significantly lower (*p* < 0.001) than population norms [12], while mental scores were not (*p* = 0.073). This finding aligns with previous studies observing preserved mental scores at 12-months post-operatively [16, 17]. Therefore, it appears that mental QoL may be more resistant to change, possibly due to psychological adaptation and survivor resilience [18].

While not an official question, most patients expressed that they would elect to undergo exenteration again, suggesting a favourable retrospective perception of the QoL trade-offs. This finding should be considered with caution given that it may be influenced by choice-supportive bias.

This study was among the first to examine the direct relationship between pain and QoL in an exenteration cohort. High pain intensity and disability were associated with poorer physical and mental QoL, with these effects persisting increasing pain grades. A subgroup analysis of patients without pain (*n* = 12) revealed physical and mental QoL scores comparable to population norms (*p* = 0.534; *p* = 0.634). These results appear to suggest that effective pain management may partially restore physical QoL. However, those who report higher pain severity tend to have greater rates of comorbidity and clinical complexity, meaning a simple bivariate relationship between pain and QoL may oversimplify their true association. Future studies should employ multilinear regression to determine potential collinearity and adjust for covariates.

The mismatch between relatively preserved disability scores and reduced physical QoL suggests that functional impairment extends beyond pain. Other factors such as stoma care, fatigue and loss of autonomy (both socially and personally) were highlighted as challenges from the patient-reported outcome measures and thematic descriptions.

### Predictors of pain

The persistence of pain highlights the importance of considering pre-, peri and post-operative factors as potential contributors to pain. Previous studies have typically limited their investigation to pre- and peri-operative prognostic factors such as bone resection, cancer type, surgical type and resection margin [5, 10].

Of the analysed factors, post-operative cancer recurrence and/or metastasis was associated with a significant increase in the likelihood of pain (*p* = 0.044), with the entirety of the subgroup reporting pain. Pre-operative recurrent malignancies also trended towards significance (OR = 7.00, CI 95% [0.81, 60.33], *p* = 0.073). The emergence of recurrence as both a pre-operative and post-operative contributor may indicate that disease recurrence represents a key risk factor for pain in exenteration populations.

No other significant associations were identified, likely due to the small cohort and binary categorisation of data, which reduced statistical power and widened 95% confidence intervals. In contrast to previous research, our study did not identify total exenterations, sacrectomies (bony resections) or nerve resections as increasing pain odds [5, 17, 19]

Pelvic complications are common in pelvic exenteration cohorts [20] and have been associated with a twofold increased pain risk in general surgical populations [21, 22]. This may partly explain the prevalence of pain, as 77% of participants experienced a surgical and/or in-patient complication. This highlights the need for incorporating complication prevention and management in pain reduction strategies.

After discharge, there was a high incidence of reported ongoing pelvic issues, particularly hernias (46%), recurrent urosepsis (40%) and pelvic collections (33%). Pelvic collections are of particular clinical interest given their previous reported link to pain shown by Wong et al. [23]. Furthermore, the long-term efficacy of current management strategies (drainage and surgery [24]) remains contentious [22], with several patients in this cohort experiencing recurrent collections associated with ongoing pain. The role of pelvic collections remains difficult to ascertain, as their aetiology may represent an infection, malignancy or a common benign feature of recovery.

Additionally, 67% of patients required additional surgery to manage ongoing complications, recurrence and comorbidities. Repeat operations may lead to wind-up and potentiation of pain [25] due to surgical complexity, scar tissue and inflammation, thereby increasing the odds of chronic pain (OR = 1.62, 95% CI [0.42, 6.26]). Although not statistically significant (*p* = 0.500) in this instance, the number of patients requiring additional surgery represents an avenue for further investigation.

### Pain management

At discharge, most patients (85%) were prescribed opioids, while current opioid prescription rates were 25%. OME was significantly lower across time points (*p* < 0.001), which likely reflects effective opioid weaning in most patients, but also possibly opioid hesitancy, as previously identified by O’Dell et al. [26]. The proportion of patients discharged with opioids was notably higher than previous rates, while neuropathics (gabapentin and pregabalin) were relatively similar [17]. This disparity in requirements may be a consequence of the high surgical/inpatient complication rate in or prescribing differences across sites.

In relation to current management, paracetamol was the most common analgesic (69%). Yet, median pain relief gained from medication was 65%, indicating potential room for optimisation and the limited benefit of pharmacological interventions. Allied health engagement was relatively low at 31%, possibly contributing to poorer pain outcomes given the benefits of physiotherapy in prehabilitation and rehabilitation settings in pelvic pain cases [27, 28]. Many of these patients likely engaged with Allied Health services earlier in recovery, but not long-term due to patient ineligibility and self-funding requirements [29]. Several patients expressed a lack of awareness of the available services, their use and potential benefit. In addition, only 17% of patients were currently seeing a pain specialist. While this appears inconsistent with the high incidence of pain in the cohort, it more closely reflects the number of patients currently utilising opioids (25%). Although there were high rates of GP engagement (92%), which likely included pain management, these findings may indicate the opportunity for more referrals to pain teams specialised in managing these complex cases. These findings suggest limited access to and efficacy of pharmacological and non-pharmacological pain management strategies. Standard practice should focus on reframing pain management goals and supporting patients in long-term self-management to improve QoL despite chronic pain.

### Strengths, limitations and future research

This study had several limitations. Despite a respectable recruitment rate of 43%, there was a small sample size, limiting the statistical power of our analysis. As a survivor analysis, selection bias may have been introduced through the inclusion of patients with more favourable outcomes. Its retrospective design resulted in reporting inconsistencies and missing data (3.50%), necessitating data sourcing from multiple locations. While the utilisation of general patient-reported outcome measures (CPGS and SF-12) remains standard in exenteration studies, it lacks specificity, limiting the clinical applicability and comparability with existing and emerging literature. To compensate, exenteration-specific questions were asked during the telephone survey. Future research would benefit from the development of a novel and validated exenteration-specific instrument [30] that includes specific questions relating to chronic pain. Nonetheless, patient-reported outcome measures remain susceptible to recall and response-shift bias.

This study included patients at the longest interval since pelvic exenteration with a direct focus on pain, including those up to 13 years post-surgery. It addresses a key gap by extending analysis beyond 5-year survivorship and recurrence rates. Importantly, the research adopted a patient-centred approach, providing a unique perspective into post-operative outcomes for prospective candidates. A mixed-methods study would be beneficial to provide deeper insight into the unique qualitative experiences of pelvic exenteration patients.

A multicentre prospective longitudinal study is recommended to generate a larger sample, to better patient engagement and to reduce the risk of extreme-response biases. This design would also address missing data and survivor bias, both of which may have skewed the results. Future directions should seek to incorporate predictive regression analyses of pre-, peri- and post-operative factors to aid the optimisation of patient outcomes.

## Conclusion

In conclusion, chronic pain prevalence in this patient cohort was high (75%), with pain persisting well beyond standard surgical recovery periods (mean = 6.10 ± 3.55 years). Most patients reported either none (25%) or low-intensity pain (29%). Both higher pain intensity and disability were associated with poorer QoL outcomes, underscoring the importance of targeting pain management in efforts to improve QoL. The heterogeneity in patient characteristics, pain and QoL outcomes highlights the need for targeted and personalised long-term clinical management. Despite sample size limitations (*N* = 48), metastasis and/or recurrence in a post-operative setting was identified as a potential predictor of chronic pain. This study placed pain at the centre of its focus, providing the foundation for a future prospective, longitudinal, multicentre study examining prognostic factors across the cancer journey to guide future clinical management.

## Supplementary Information

Below is the link to the electronic supplementary material.

ESM 1 (1.28 MB DOCX)

## Data Availability

The data is stored in a password-protected REDCap database in accordance with site specific data governance guidelines at the Peter MacCallum Cancer Centre in accordance with site specific guidelines. Requests for access to the raw data are subject to institutional approval.
