# Supplementary material for: Patient experience after pelvic exenteration: chronic pain and quality of life
Source: Support Care Cancer. 2026 Jun 15;34(7):660. doi: 10.1007/s00520-026-10848-y (PMC13269298; doi:10.1007/s00520-026-10848-y)
Supplement: Supplementary file 1 — (1.28 MB DOCX) [file 520_2026_10848_MOESM1_ESM.docx]

Supplementary Figures and Tables

| ***Table S1: Comparison of QoL Between the Pelvic Exenteration Cohort and Populations Norms*** | | | | | | |
| --- | --- | --- | --- | --- | --- | --- |
|  | Population Norm | Test | Test Statistic | *p* | Effect Size | 95% CI Effect Size |
| Physical | 50.12 | One sample *t* | *t* (47) = −6.09 | < 0.001 | *d* = −0.88 | [−1.21, −0.54] |
| Mental | 52.85 | Wilcoxon signed- rank | *z* = −1.80 | 0.073 | *r* = −0.26 | N/A |

*Note.* 95% CI = 95% confidence intervals

# Appendix

**Appendix i. Patient Telephone Survey**
